# Shikonin induces programmed death of fibroblast synovial cells in rheumatoid arthritis by inhibiting energy pathways

**DOI:** 10.1038/s41598-021-97713-6

**Published:** 2021-09-14

**Authors:** Jiahui Li, Jinglong Pang, Zhe Liu, XianMing Ge, Yanan Zhen, Chen Chen Jiang, Yaming Liu, Qiang Huo, Yiming Sun, Hao Liu

**Affiliations:** 1 School of Pharmacy, Bengbu Medical College, Donghai Road, Bengbu, 233030 Anhui China; 2 The First Affiliated Hospital of Bengbu Medical College, Zhihuai Road, Bengbu, 233000 Anhui China; 3 Cancer Neurobiology Group, School of Biomedical Sciences and Pharmacy, The University of Newcastle, Callaghan, NSW 2308 Australia; 4 Hunter Medical Research Institute, New Lambton, NSW 2305 Australia

**Keywords:** Biological techniques, Immunology, Molecular biology, Molecular medicine, Rheumatology

## Abstract

Shikonin is the main component of the traditional Chinese medicine comfrey, which can inhibit the activity of PKM2 by regulating glycolysis and ATP production. Rheumatoid arthritis synovial cells (RA-FLSs) have been reported to increase glycolytic activity and have other similar hallmarks of metabolic activity. In this study, we investigated the effects of shikonin on glycolysis, mitochondrial function, and cell death in RA-FLSs. The results showed that shikonin induced apoptosis and autophagy in RA-FLSs by activating the production of reactive oxygen species (ROS) and inhibiting intracellular ATP levels, glycolysis-related proteins, and the PI3K-AKT-mTOR signaling pathway. Shikonin can significantly reduce the expression of apoptosis-related proteins, paw swelling in rat arthritic tissues, and the levels of inflammatory factors in peripheral blood, such as TNF-α, IL-6, IL-8, IL-10, IL-17A, and IL-1β while showing less toxicity to the liver and kidney.

## Introduction

Rheumatoid arthritis (RA) is a systemic autoimmune disease with chronic, symmetrical, and multiple synovial arthritis and abarticular pathological changes in the primary clinical manifestation. The rate of disability is exceptionally high in the later period. The pathogenesis of RA is mediated by synovial tissue macrophages and fibroblast-like synoviocytes (FLSs), which are characterized by joint injury, inflammation and destruction. It has been reported that T cells play an essential role in the progression of RA, and synovial fibroblasts have become a necessary participant in the development of RA^1–3^. RA exhibits an aggressive phenotype that causes synovial inflammation and cartilage damage due to inflammatory mediators^4^. Although the role of synovial fibroblasts in arthritis has been acknowledged, their functional molecular mechanisms remain unclear.

In recent years, the clinical diagnosis and treatment of RA have focused on standard individualized treatment^5^. Currently, RA treatments include disease-modifying drugs, such as anti-rheumatic drugs (DMARDs), nonsteroidal anti-inflammatory drugs (NSAIDs), and glucocorticoids. Although the treatment of RA has improved, currently available disease-modulating drugs cannot directly target FLS dysfunction. Therefore, new and rational drugs are required to replace or supplement current therapies.

Shikonin is a natural dye and food additive with antiviral^6^, antioxidant^7^, anti-inflammatory^8^, accelerated wound healing, and immunity-enhancing properties^9^. Because of these advantages, shikonin has been widely used in recent years. Studies have shown that shikonin can effectively inhibit PKM2 but has no effect on PKM1 or pyruvate kinase-L (PKL), thereby regulating glycolysis and ATP production^10^. Shikonin has been shown to induce apoptosis in various cancer cells, including the HL60 human promyelocytic leukemia cell line, liver cancer, prostate cancer, colorectal cancer, oral squamous cell carcinoma, basal cells, osteosarcoma, HeLa cervical cancer cells, and the T24 bladder cancer cell line^11–19^. Interestingly, PKM2 expression in RA-FLSs is three times higher than that in normal FLSs^20^. Although the ROS scavenging and anti-inflammatory activity of shikonin have been reported in RA-FLSs^21–24^, few studies have elucidated the mechanism by which shikonin induces RA-FLS cell death. The purpose of this study was to investigate the role of shikonin in inducing programmed death of fibroblast synovial cells in rheumatoid arthritis by inhibiting energy pathways.

In our study, shikonin induced apoptosis and autophagy of RA-FLSs by activating the production of ROS and inhibiting intracellular ATP levels, glycolysis-related proteins, and the PI3K-AKT-mTOR signaling pathway. Shikonin can significantly reduce the expression of apoptosis-related proteins, paw swelling in rat arthritic tissues, and the levels of inflammatory factors in peripheral blood, such as TNF-α, IL-6, IL-8, IL-10, IL-17A, and IL-1β.

## Materials and methods

### Reagents and antibodies

Human RA-FLSs and FLSs were purchased from the Chinese Academy of Sciences, Shanghai. Dulbecco’s modified Eagle’s medium (DMEM) and fetal bovine serum (FBS) were purchased from Gibco, USA. Penicillin (100 U/ml) and streptomycin (100 mg/l) were used for all cell culture experiments. Shikonin and methotrexate (MTX) were purchased from Maclean Biochemical Technology, Shanghai. MTT and complete Freund’s adjuvant (CFA) were purchased from Sigma, USA. Antibodies against Bcl-2, Bax, cleaved caspase 3, LC3, p-Akt, mTOR, PI3K, PKM2, GLUT1, HK2, and β-actin were purchased from Abcam. HRP-labeled goat anti-rats IgG goat anti-rabbit IgG was purchased from Boster Bioengineering. ATP kits, mitochondrial membrane potential detection kits (JC-1), reactive oxygen kits, and lactic acid kits were purchased from Biyuntian. An Annexin V/PI double staining kit was purchased from Biobox. Sprague–Dawley (SD) male rats were purchased from the animal center of Bengbu Medical College. Aspartate aminotransferase (AST/GOT), alanine aminotransferase (ALT/GPT), and creatinine kits were purchased from Jiancheng Reagent, Nanjing. ELISA kits for TNF-α, IL-6, IL-8, IL-10, IL-17A, and IL-1β were purchased from Kaiji Biotechnology, Jiangsu. The reagents used for RT-qPCR were purchased from Thermo Scientific, USA. The primers were purchased from General Biosystems, Anhui. Lianchuan Biological, Hangzhou, provided the gene chip.

### Cell culture

RA-FLSs and FLSs were cultured in Dulbecco’s modified Eagle’s medium (DMEM) supplemented with fetal bovine serum (FBS), 80 U/ml penicillin and 100 U/ml streptomycin. Cells were grown in an atmosphere of 5% CO_2_ at 37 °C.

### MTT assay

RA-FLSs and FLSs were seeded in a 96-well plate at a density of 5 × 10^3^ cells/ml and cultured for 24 h at 37 °C with 5% CO_2_. After 24 h of incubation, cells were exposed to different shikonin concentrations (2, 2.5, 3 µmol/l); cells were further maintained in the incubator for 24 h, 48 h, or 72 h at 37 °C with 5% CO_2_. Sequentially, 15 μl of MTT solution (5 mg/ml) was added to each well. After 4 h, the supernatant was aspirated, 150 μl of dimethyl sulfoxide (DMSO) was added to each well followed by incubation at 37 °C for 30 min to completely dissolve the formazan. The absorbance value was measured by enzyme-linked immunosorbent assay (ELISA) at a wavelength of 490 nm. Cell viability (%) = (A test group − A control control)/(A blank control − A control) × 100%.

### Cell morphology

RA-FLSs in the logarithmic growth phase were prepared as single-cell suspensions and seeded in 6-well plates at a density of 2 × 10^5^/well. The cells were cultured in an incubator for 24 h; cells were then exposed to different shikonin concentrations. Morphological images of the cells were captured using an inverted microscope (Nikon Eclipse TS200, Tokyo, Japan).

### Transmission electron microscopy

RA-FLSs were seeded in 6-well plates (1 × 10^6^ cells per well) and cultured for 24 h. The control group cells were cultured with DMSO, and the shikonin group cells were cultured with 3 µmol/l shikonin. After 24 h of continuous culture, the cells were harvested by centrifugation. The cells were fixed, stored at low temperature, sent to Saville for follow-up treatment, and observed by transmission electron microscopy (TEM).

### Annexin V-FITC/PI double staining

RA-FLSs and FLSs were prepared into single-cell suspensions and seeded in 6-well plates at 2 × 10^5^ cells per well. Cells were treated with shikonin for 24 h; cells were collected and centrifuged at 1200 r/min for 5 min. Buffer-suspended cells (500 μl) were added with 10 μl of Annexin-V-FITC, mixed at room temperature for 15 min, and 5 μl of PI was added.

### JC-1 staining

RA-FLSs were prepared as single-cell suspensions, seeded in 12-well plates at 1 × 10^5^ cells per well with different shikonin concentrations (2, 2.5, 3 μmol/l) and cultured for 24 h. After 48 h, the culture solution was aspirated and replaced with 1 ml of the cell culture media. The cell culture supernatant may contain serum and phenol red. One milliliter of JC-1 staining solution (JC-1 solution: ultrapure water = 1:1000) was added followed by incubation in a cell incubator at 37 °C for 20 min. After incubation, the supernatant was aspirated and the cells were washed twice with JC-1 staining buffer. Two milliliters of cell culture medium was added and then the cells were observed under a fluorescence microscope for image capture.

### Intracellular ATP level

RA-FLSs were seeded in 6-well plates (2 × 10^5^ cells/well) for 24 h. Cells were treated with different shikonin concentrations (2, 2.5, 3 μmol/l) for 6 h. The cells were collected by centrifugation, and the supernatant was discarded. The experiment was performed according to the manufacturer’s protocol.

### Reactive oxygen species (ROS)

RA-FLSs were seeded in 12-well plates (1 × 10^5^ cells/well) and cultured for 24 h. The cells were cultured with different shikonin concentrations (2, 2.5, 3 μmol/l), and DCFH-DA was administered after 6 h. DCFH-DA was first diluted with serum-free medium to a final concentration of 10 μmol/l. The original culture solution was removed from each well, added to 1 ml of diluted DCFH-DA, and incubated in a 37 °C cell incubator for 20 min. After washing twice with PBS, the’ fluorescence intensities of the treated groups at different shikonin concentrations were observed with a fluorescence microscope.

### Lactic acid assay

RA-FLSs were seeded in 6-well plates at 2 × 10^5^/well and incubated for 24 h. Shikonin (0, 2, 2.5, 3 μmol/l) was added for 24 h. Cells were collected and resuspended in an appropriate amount of lysate and transferred to an EP tube. The cells were lysed three times at − 20 °C and centrifuged at 12,000 r/min for 30 min at 4 °C. The supernatant was used for subsequent analysis. According to the lactic acid assay kit (BioVision), the experiment was repeated 3 times as described.

### Western blotting

RA-FLSs were treated with different concentrations of shikonin for 24 h and then collected. Total cell lysates were prepared using RIPA buffer supplemented with protease inhibitors (Roche, Shanghai, China) and PMSF (Sigma, USA). Protein concentrations were determined with BCA kits (Beyotime, Shanghai, China). The cells were separated by 10% SDS-PAGE and transferred to PVDF membranes (Millipore, Billerica, MA, USA). After blocking with 5% skim milk, the PVDF membranes were incubated with their specific primary antibodies in TBST at 4 °C with primary antibodies recognizing PKM2 (1:1000, Cell Signaling Technology, USA), GULT1, HK2, PI3K, p-PI3K (1:1000, Santa Cruz Biotechnology, USA), AKT, p-AKT (1:1000, Abcam, USA), mTOR, BAX (1:1000, Cell Signaling Technology, USA), Bcl-2 (1:1000, Cell Signaling Technology, USA), caspase 3 (1:1000, Enzo, USA), LC3 (1:1000, Santa Cruz Biotechnology, USA), and anti-β-actin (1:1000, Biosharp, China). All reagents were dissolved according to the manufacturer’s instructions.

After three washes with TPBS, the secondary antibody (1:5000) was added followed by incubation at room temperature for 1 h. Proteins were visualized and detected by enhanced chemiluminescence detection reagents (Pierce, Thermo Fisher Scientific) and analyzed with an Image Quant LAS 4000 imaging system (GE Healthcare, Pittsburgh, PA, USA).

### Blue Native PAGE, BN-PAGE

After 48 h of shikonin treatment, the cell supernatant was aspirated and discarded, and the cells were washed with PBS twice. Proteinase and phosphorylase inhibitors were added to the BN-PAGE protein lysate (50 mM BisTris-HCl, 0.5 M 6-amino-caproic acid, 10% glycerol and 1% digitalin, pH 7.0) to decompose on ice for 30 min followed by centrifugation at 16,000*g* at 4 °C for 15 min. Protein concentrations were determined with BCA kits (Beyotime, Shanghai, China), and then loading buffer was added to prepare a BN-PAGE protein sample. The prepared protein sample was loaded, and inner tank electrophoresis solution (0.05 M Tricine, 15 mM BisTris, pH 7.0) and the outer tank electrophoresis solution (0.05 M BisTris-HCl, pH 7.0) were added for 100 V constant pressure electrophoresis. After membrane transfer at a constant current of 300 mA, the PVDF membrane was decolorized with methanol and washed twice with TBST, and 10% skimmed milk was added for blocking. The subsequent steps were the same as those for Western blotting. All antibodies are shown in supplementary 1.

### ELISA assay

Rats were treated with shikonin at 1 mg/kg and 2 mg/kg for 32 days, and peripheral blood was collected from the abdominal aortas of each group (n = 6). Peripheral blood was centrifuged at 12,000*g* for 10 min at 4 °C, and the supernatant was analyzed for the contents of TNF-α, IL-6, IL-8, IL-10, IL-1β, and IL-17A. The required reaction plate was removed, and 10 µl of standards and 10 µl of each specimen were added to the corresponding reaction plate wells. Then, 40 µl of anti-rats IL-1 biotin and 40 µl of anti-rats IL-1 POD was added to each well, followed by gentle mixing for 30 s and incubation at room temperature for 45 min. the plate's liquid was shaken off, the reaction plate was washed with washing solution (by adding 350 µl of washing solution to each well), and the water droplets were removed. These washes were repeated 5 times. Next, 100 µl of color developing solution was added to each well, followed by gentle mixing for 10 s and incubation at room temperature for 20 min. Finally, 100 µl of stop solution was added to each well, followed by gentle mixing for 30 s, and the OD value at 450 nm was measured within 30 min. The concentration was determined from the standard curve line according to the OD value of the sample.

### RT-qPCR

After shikonin treatment, the cell supernatant was aspirated and discarded, and the cells were washed with PBS twice. Total RNA was isolated using an RNeasy mini kit (QIAGEN, Beijing, China). Complementary DNA (cDNA) was synthesized using a high capacity cDNA reverse transcription kit (Thermo, Shanghai, China). Real-time fluorescence quantitative PCR was performed on cDNA, and a 10 μl reaction system was prepared: cDNA, upstream and downstream primers (0.5 μl), SYBG (5 μl) (04913914001, Roche) and DEPC to make up to 10 μl. The reaction conditions were as follows: 95 °C 10 min; Reps 40: 95 °C 15 s, 60 °C 1 min; 95 °C 15 s; 60 °C 1 min; 95 °C 15 s. All primers are shown in supplementary 2. All reactions were repeated three times.

### PKM2 siRNA knockdown

Cells were transfected with oligo small interfering RNAs (siRNAs) using Lipofectamine 2000. The PKM2 sequence: 5′-CCAUAAUCGUCCUCACCAAUU-3′ and control sequence: 5′-UUCUCCGAACGUGUCACGUTT-3′ were purchased from Jima, Shanghai. Transfection conditions that yielded ≥ 70% knockdown after 48 h, as determined by Western blotting with minimal toxicity, were used in the study.

### Gene chip

RA-FLSs were prepared into a single-cell suspension and seeded in large dishes. Shikonin was used for treatment at a concentration of 3 µmol/l for 24 h, and cells were collected by centrifugation. Cells were lysed with TRIzol solution for RNA extraction. After the total RNA passed the quality inspection, the magnetic beads connected with Oligo (dT) enriched the eukaryotic mRNA. The extracted mRNA was randomly broken into short fragments by a fragmentation reagent. Using the fragmented mRNA as a template, a six-base random primer was used to synthesize single-strand cDNA, and buffer, dNTPs, RNase H, and DNA Polymerase I were added for stranded two-cDNA. AMPure XP beads purified the double-stranded products and T4 DNA polymerase and Klenow DNA polymerase activities repaired the sticky ends of DNA. AMPure XP beads selected the fragments and finally PCR amplification was performed to increase the final sequencing library. After the library quality inspection was qualified, an Illumina HiSeq4000 was used for sequencing (length was 2 × 150 bp, PE150).

### Preparation of the adjuvant arthritis (AA) animal models

Six-week-old male Sprague–Dawley rats (SCXK 2019-0002) weighing approximately 160-180 g were purchased from the Laboratory Animal Center of Zhejiang Academy of Medical Sciences and fed in the Laboratory Animal Center of Bengbu Medical College at room temperature. Animal experiments followed institutional guidelines and were approved by the Bengbu Medical College Institutional Animal Care and Use Committee with protocol reference No. 2017038. In addition, animal experiments followed institutional ARRIVE guidelines. Complete Freund's adjuvant (CFA) was removed from the refrigerator and mixed. Rats in the AA group and the experimental group were routinely sterilized. Their right limbs were straightened, and 0.1 ml of CFA was subcutaneously injected into the middle of the right hind toe. Each rat was injected with 0.1 ml of CFA (referred to as an AA rat). Animals were randomized and divided after surviving the initial treatment using a computer-based random order generator. The control group was injected with 0.1 ml of normal saline. After 14 days, the rats were randomly divided into five groups: control group, AA group, MTX group (0.5 mg/kg/3 day), low-dose shikonin group (1 mg/kg/day) and high-dose shikonin group (2 mg/kg/day). The left hind paw volume of each rat was assessed as follows: the average volume was taken as the standard foot volume (Vn) before inflammation. On the 14th day of inflammation, the rats were intragastrically administered shikonin according to the weight of each rat. The control group and the AA group were administered normal saline intragastrically for 20 days, n = 6.

### Detection of toe swelling

From the 14th day of the experiment, the left hind toe volume was recorded every 3 days. The volume change of the left posterior toe in each group was calculated as follows: the volume of hind paw swelling (Δml) = V_t_ − V_0_; V_0_ = the volume before CFA immunization (ml) and V_t_ = the volume at day t after CFA immunization (ml).

### Liver and kidney toxicity assay

Blood (2–4 ml) was extracted from the inferior vena cava of the rats and placed at rest for 2 h. The blood was then centrifuged at 2500 r/min for 10 min. The serum was separated and stored at − 80 °C. The experiment was performed according to the manufacturer’s protocol as described for ALT, AST, and Cr.

### Immunohistochemistry

Dewaxing and rehydration of paraffin-embedded paw sections were performed using xylene and then degreased with gradient alcohol. Hydrogen peroxide (3%) at room temperature was used to fix the samples for 10 min and block them with blocking solution for 30 min. The primary antibody (1:50) was incubated at 37 °C for 60 min and stained with the secondary antibody. Samples were incubated for 10 min at room temperature and developed with DAB. Secondary staining of the nucleus was performed with 1% hematoxylin. Antibodies against the following proteins were used: Bcl-2 and Bax (Cell Signaling Technology, USA) and caspase 3 (Enzo, USA). All reagents were dissolved according to the manufacturer’s instructions.

### Joint histopathology

On the 32nd day after establishing the model, the rats were anesthetized, and the inflammatory synovial tissue was removed. The joint samples were fixed in 40 g/l paraformaldehyde, decalcified with 100 g/l nitric acid, washed with water, and embedded in paraffin for H&E staining. Pathological histological changes were observed under a light microscope and photographed.

### Statistical analysis

The experimental results were analyzed by SPSS 21.0 software. The experimental data are expressed as the mean ± s. One-way ANOVA and LSD tests were used to compare the differences between the groups. *p* < 0.05 was considered statistically significant.

## Results

### Effect of shikonin on energy metabolism in RA-FLSs

**Figure 1:**
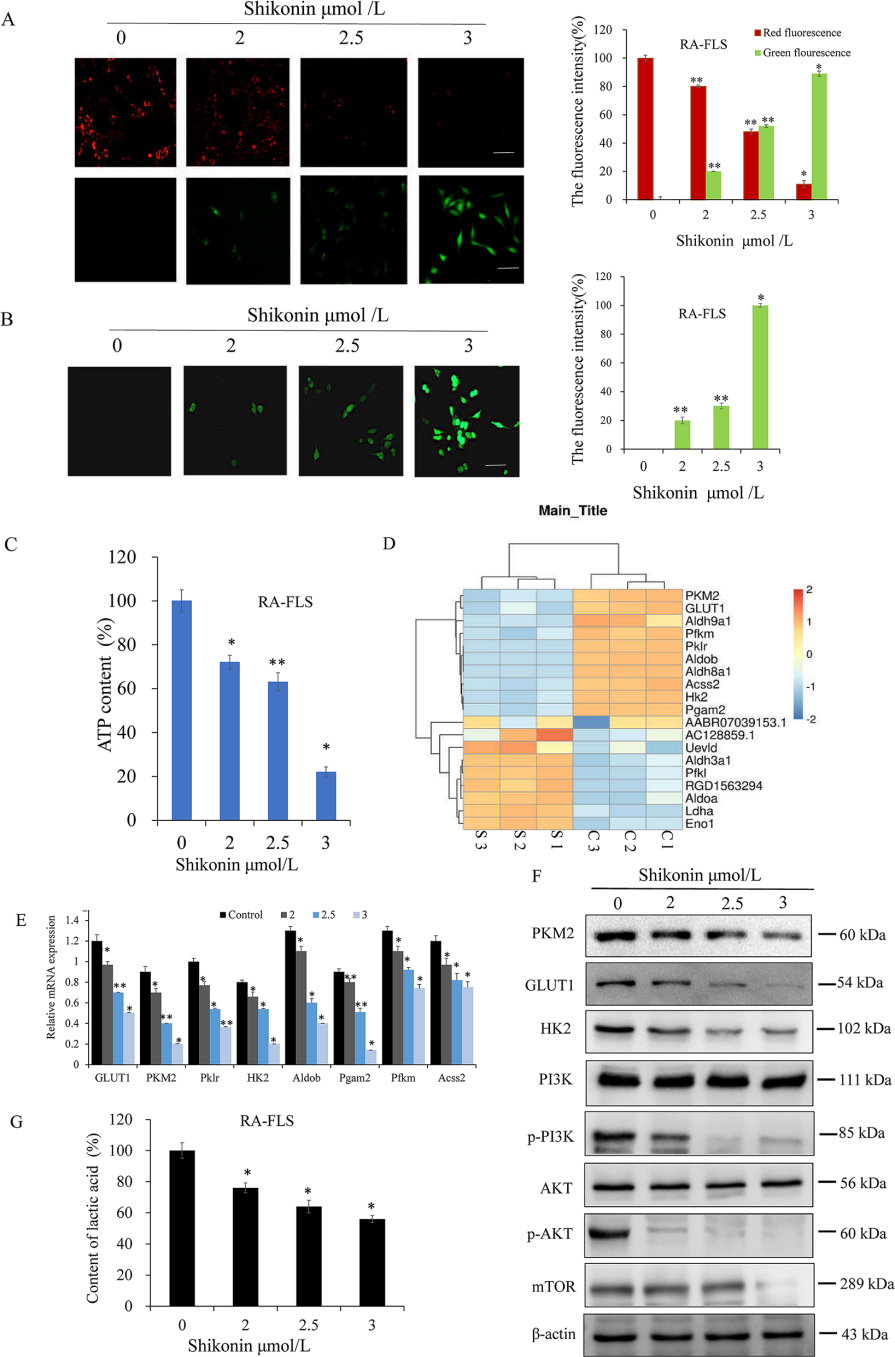
Effects of shikonin induction on energy metabolism in RA-FLSs. (**A**) The mitochondrial membrane potential of RA-FLSs after adding increasing concentrations of shikonin for 24 h (red indicates normal mitochondrial membrane potential). (**B**) Intracellular ROS changes in RA-FLSs at different concentrations of shikonin after 6 h. (**C**) Intracellular ATP changes detected using an ATP kit in RA-FLSs treated with different concentrations of shikonin for 6 h. (**D**) Genes related to glycolysis with a larger difference compared to the control group that were selected from the gene chip results. (**E**) RT-qPCR verified the above differentially expressed genes. (**F**) Western blot showing the protein levels of glycolysis-related proteins and related signaling pathways in RA-FLSs treated with different concentrations of shikonin for 24 h. (**G**) Intracellular lactic acid changes detected by using a lactic acid kit. *p < 0.05, **p < 0.01 vs Control group. Scale bar represents 200 μm. Data are representative of at least three independent experiments. *S* Shikonin, *C* Control.

RA-FLSs are capable of supplying energy through glycolysis and mitochondrial oxidative phosphorylation. We first examined the effects of shikonin on the mitochondrial function of RA-FLSs by assessing reactive oxygen species and intracellular ATP levels. Shikonin (for 24 h) significantly reduced the mitochondrial membrane potential of RA-FLSs (Fig. 6B). 1A). Red fluorescence from JC-1 indicated standard mitochondrial membrane potential, whereas green fluorescence demonstrated diminished mitochondrial membrane potential and therefore indicated that the cells were an early stage of apoptosis. The ROS assay results showed that green fluorescence gradually increased with increasing shikonin concentration (Fig. 6B). 1B), suggesting that shikonin can induce a large increase in ROS in RA-FLSs. After administration, the intracellular ATP changes were measured, and RA-FLSs were treated with shikonin for 6 h. Compared with the control group, shikonin induced ATP in RA-FLSs, with ATP levels reduced from 100% at baseline to 72%, 63%, and 22% (Fig. 1C). Subsequently, the effect of shikonin on glycolysis in RA-FLSs was evaluated. First, the gene chip was screened for glycolysis-related genes, including PKM2, GLUT1, HK2, Pklr, Aldob, Pgam2, Pfkm, and Acss2, and the diclofenac sodium group genes, including PKM2, GLUT1, and HK2, were found. Their expression was most significant (Fig. 1D). After that, the above differentially expressed genes were verified by RT-qPCR and WB. The expression of glycogen proteins PKM2, GLUT1, and HK2 decreased with increasing concentrations of shikonin (Fig. 1E,F). Inhibition of the PI3K/AKT/mTOR pathway (Fig. 1F) and inhibition of lactic acid, which is the final product of glycolysis (Fig. 1G), further validated that shikonin can inhibit RA-glycolysis in FLSs. These results indicate that shikonin can inhibit energy metabolism in RA-FLSs by inhibiting glycolysis and reducing mitochondrial function.

### PKM2 plays a critical role in energy metabolism in RA-FLSs

**Figure 2:**
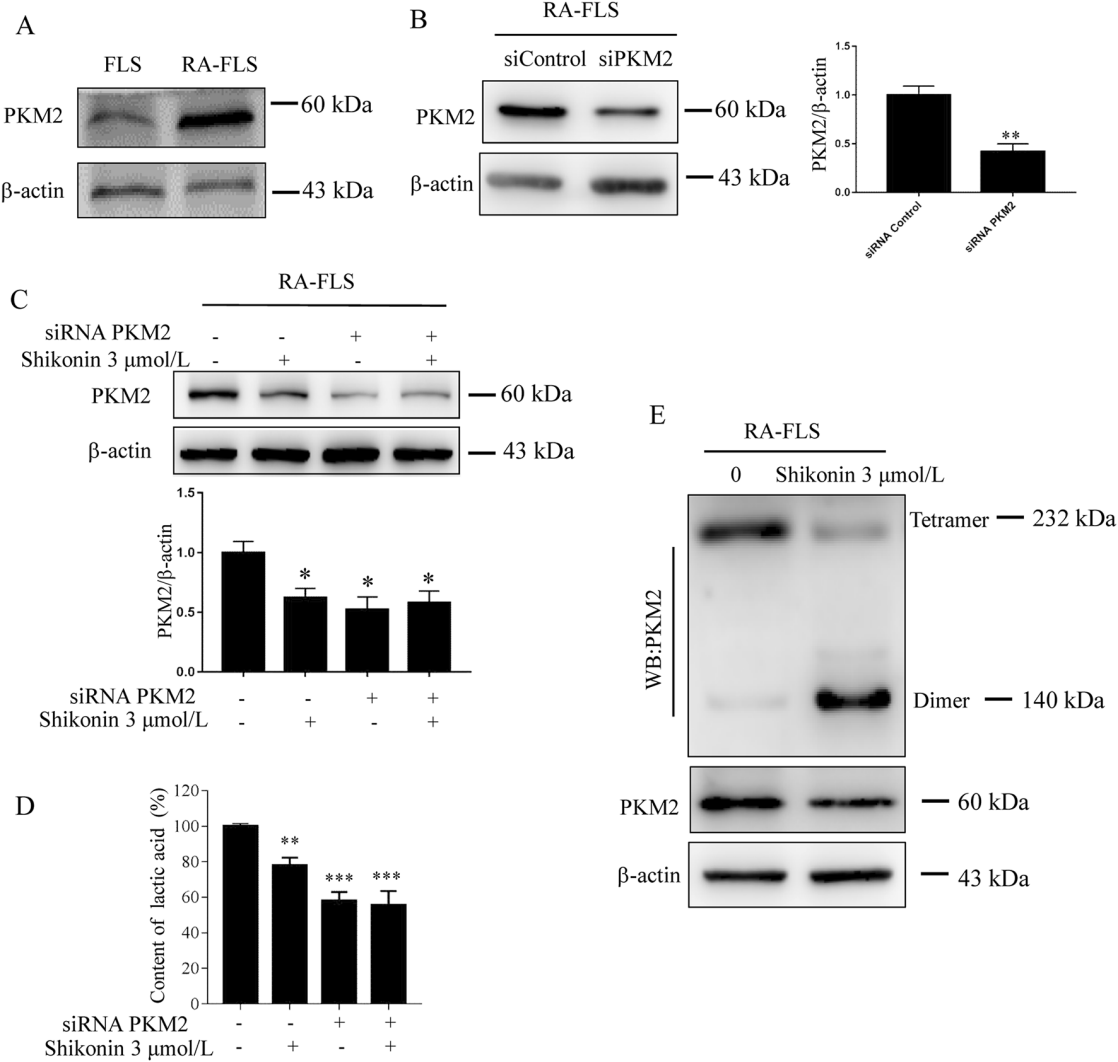
PKM2 plays a critical role in energy metabolism in RA-FLSs. (**A**) Western blot analysis of PKM2 protein expression in RA-FLSs and FLSs. (**B**,**C**) Western blot analysis of PKM2 protein expression in RA-FLSs treated as indicated (siRNA Control, siRNA PKM2). (**D**) Intracellular lactic acid changes detected by using a lactic acid kit when PKM2 was knocked down in RA-FLSs. (**E**) PKM2 tetramer vs*.* dimer formation after shikonin exposure for 24 h in RA-FLSs. *p < 0.05, **p < 0.01 vs Control group. Data are representative of at least three independent experiments.

The gene chip results showed that PKM2 was the most significant change in RA-FLSs. The WB results also showed that the expression of PKM2 in RA-FLSs was higher than that in RA-FLSs (Fig. 2A). To verify this conclusion, PKM2 expression was knocked down in RA-FLSs (Fig. 2B), and shikonin 3 μmol/l was added for 24 h to detect PKM2 expression (Fig. 2C) and the release of lactic acid in cells (Fig. 2D). PKM2 catalyzes the upstream substrate phosphoenolpyruvate (PEP) to produce pyruvate. PKM2 often switches between its tetramer and dimer forms to determine whether glucose is converted to pyruvate for energy supply or biosynthesis. The dimer form of PKM has little affinity for PEP and acts as a brake for glycolysis. Given the importance of dimer and tetramer formation of PKM2, we performed BN-PAGE to investigate how shikonin alters PKM2 formation in RA-FLSs. RA-FLSs were treated with 3 μmol/l shikonin for 24 h. BN-PAGE studies demonstrated that there was a transition from dimer PKM2 to tetramer PKM2 (Fig. 2E). Since PKM2 dimers and tetramers have different enzymatic activities, we suggested that the mechanisms of shikonin in RA-FLSs may inhibit glycolysis and alter cell metabolism to synthesize other necessary intermediate products. The changes in PKM2 activity in RA-FLSs treated with shikonin and how shikonin regulates the level of PKM2 in cells will be further studied in the future to clarify the mechanism.

### Inhibitory effects of shikonin on the proliferation and apoptosis of RA-FLSs

**Figure 3:**
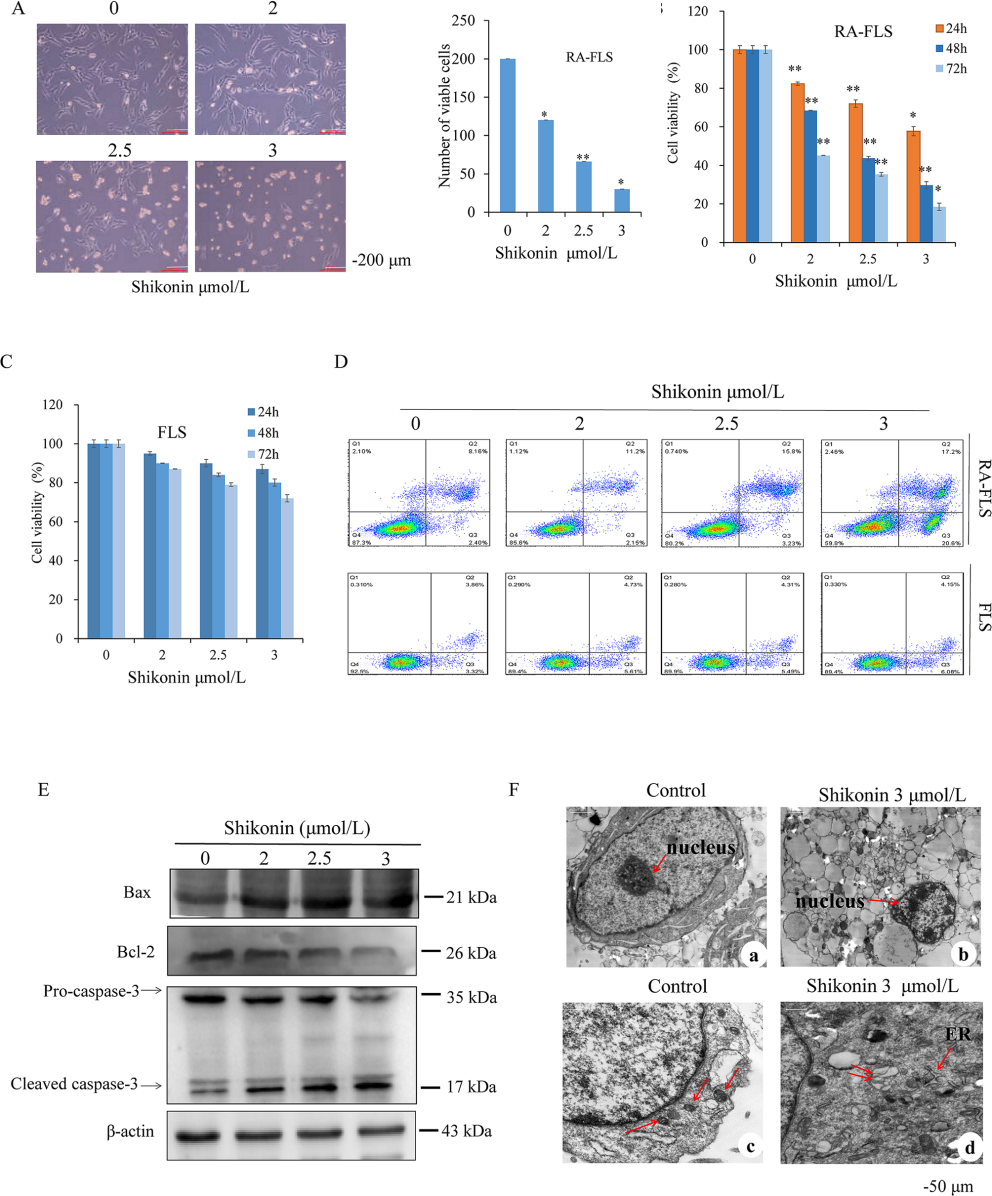
Inhibitory effects of shikonin on the proliferation and apoptosis of RA-FLSs. (**A**) Light microscopy showing the morphology of RA-FLSs. (**B**,**C**) MTT cell viability assay upon treating RA-FLSs and FLSs with shikonin for 24, 48, and 72 h. (**D**) Flow cytometry staining of RA-FLSs and FLSs with Annexin V-FITC/PI to detect RA-FLS and FLS apoptosis rates. (**E**) Apoptosis-related proteins shown in RA-FLSs by Western blot. (**F**) Transmission electron microscopy showed the submicroscopic structure of RA-FLSs from the control group and the shikonin group after 24 h of treatment with shikonin. *p < 0.05, **p < 0.01 vs the Control group. Scale bar represents 50 μm. Data are representative of at least three independent experiments.

The effect of adding different shikonin concentrations to RA-FLSs for 24 h was then assessed by microscopy. The cells showed a close association under the microscope with a fusiform structure, clearly defined membrane, and full cytoplasm. The shikonin-administered group cells showed a lower density with a higher number of suspended cells, widened cell gaps, shrunken size, unclear cell edges, and loss of normal cell morphology (Fig. 3A). The MTT assay results showed that with increasing concentrations of shikonin and extension of the action time, shikonin inhibited the proliferation of RA-FLSs but had a reduced inhibitory effect on FLSs (Fig. 3B,C). The IC_50_ values of shikonin for RA-FLSs at 24, 48, and 72 h were 3.82, 1.5, and 1.13 μmol/l, respectively, whereas those for FLSs at 24, 48, and 72 h were 6.14, 7.42, and 10.27 μmol/l, respectively. Annexin V-FITC/PI was then used to detect the apoptosis rates of RA-FLSs and FLSs. The total apoptotic rate in the RA-FLS control group was 11.49 ± 0.7% (Fig. 3D), whereas those in the 2, 2.5, and 3 μmol/l shikonin groups were 14.27 ± 2.1%, 18.08 ± 2.4%, and 38.9 ± 1.7%, respectively. Similarly, the total apoptotic rate in the FLS control group was 7.85 ± 1.1%, whereas those in the 2, 2.5, and 3 μmol/l shikonin groups were 10.4 ± 1.6%, 11.61 ± 2.0%, and 14.55 ± 2.1%, respectively. Shikonin induced the apoptosis of RA-FLSs and had little killing effect on FLSs. By transmission electron microscopy, the control group's cell bodies were found to be normal, with more organelles, clear nucleoli, smooth and intact nuclear membrane edges, and normal-looking chromatin that was evenly distributed in the nucleus. There was no apoptotic structures, such as the chromosome edge set (Fig. 3F-a). The mitochondria of the cells were clear, elliptical, or rod-shaped, with the septum arranged neatly and lacking obvious vacuoles (Fig. 3F-c). In the shikonin group, the chromatin in the nucleus was laterally shifted, concentrated, accumulated at the edge of the nuclear membrane, and had no nucleoli. Many cells showed classical characteristics of apoptosis, such as vacuole presence (Fig. 3F-b). Expansion of the endoplasmic reticulum, a decline in the number of mitochondria, swelling, shortening, and vacuolization were apparent (Fig. 3F-d). Further investigation of the possible mechanism of shikonin-induced apoptosis in RA-FLSs was undertaken. With increasing shikonin concentration, the protein levels of mitochondrial apoptosis-associated proteins such as caspase 3 and Bax increased, while the antiapoptotic protein Bcl-2 decreased (Fig. 3E), suggesting that RA-FLSs undergo apoptosis in the presence of shikonin.

### Shikonin induces autophagy in RA-FLS cells

**Figure 4:**
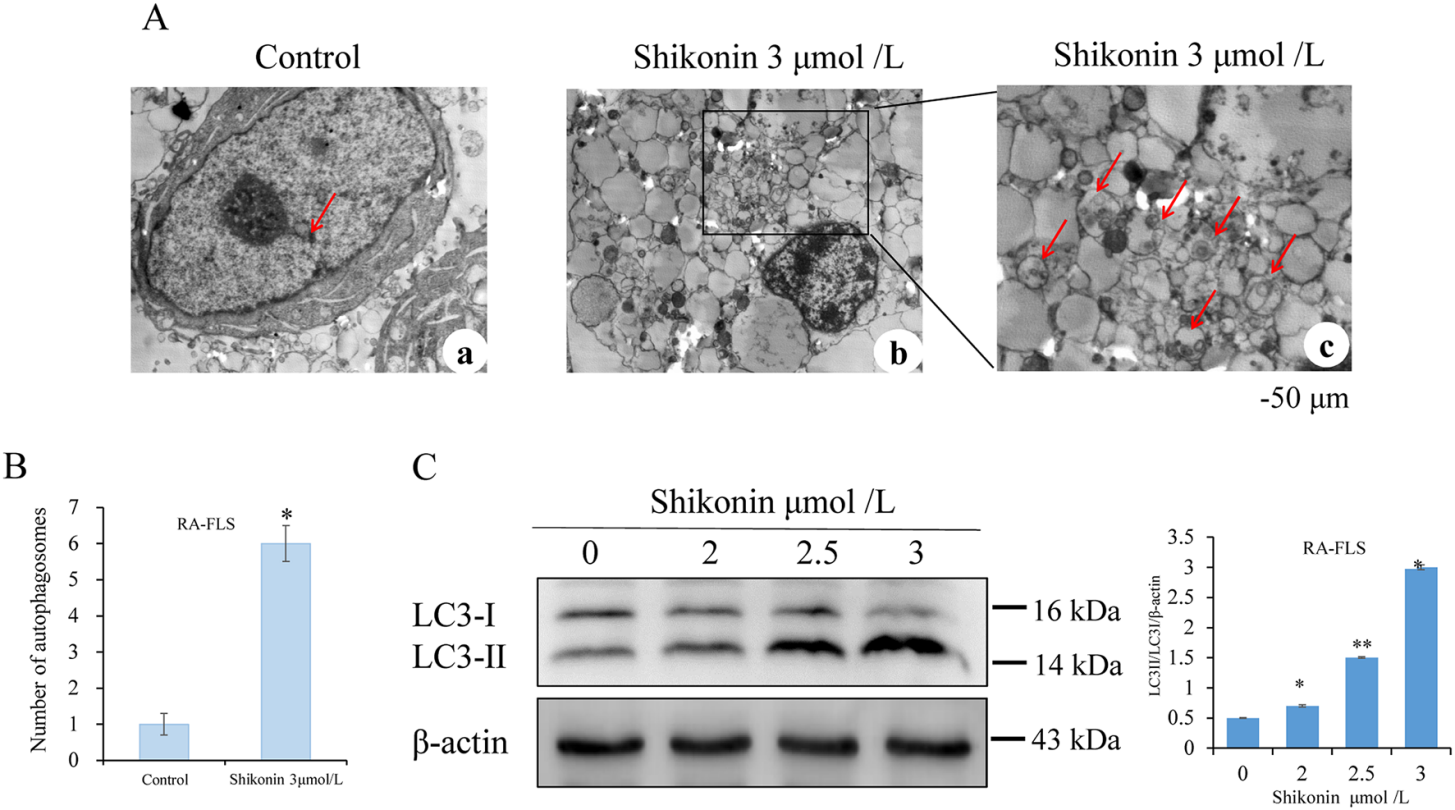
Shikonin induces autophagy in RA-FLS cells. (**A**,**B**) Transmission electron microscopy shows submicroscopic structures associated with autophagy in RA-FLSs from the control group and the shikonin group after 24 h of treatment with shikonin. (**C**) Western blot showing protein levels of LC3. Scale bar represents 50 μm. Data are representative of at least three independent experiments. *p < 0.05, **p < 0.01 vs control group.

Transmission electron microscopy showed that many autophagosomes were present in the cytoplasm of the 3 μmol/l shikonin group compared with the control group and occluded the damaged mitochondrial endoplasmic reticulum. This was characterized by the cells forming double-layered or multilayered membranes containing organelles and cytosolic components, primarily occupied by vacuoles (Fig. 4A,B). To confirm autophagy, we performed a Western blot assay. As the concentration of shikonin increased, the expression ratio of LC3-II/LC3-I increased, indicating an increase in autophagy (Fig. 4C).

### Effects of shikonin on the degree of swelling of the toes in AA rats and its effect on liver and kidney toxicity

**Figure 5:**
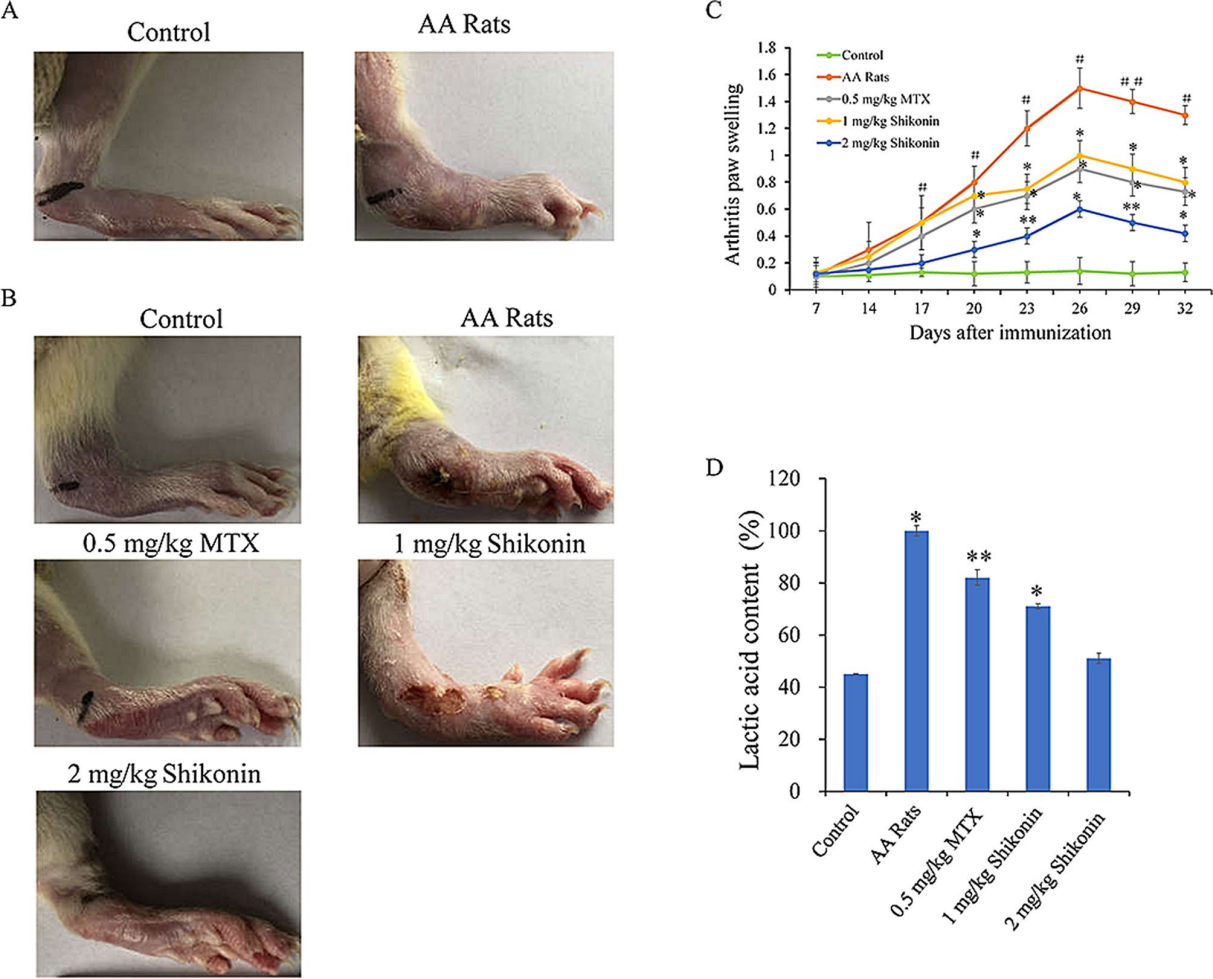
Effects of shikonin on toe swelling and hepatorenal toxicity in AA rats. (**A**) Symptoms were assessed in AA rats 14 days after immunization. (**B**) The left hind paws of rats from different groups on 32 days. (**C**) The arthritic paw swelled from days 7 to 32 in the shikonin (1 and 2 mg/kg) groups and the MTX (0.5 mg/kg) group. (**D**) Lactic acid changes were detected by using a lactic acid kit in vivo*.* (Table 1) The effects of shikonin on liver and kidney toxicity in rats. Data are expressed as the mean ± standard deviation (SD), n = 6, ^#^*p* < 0.05, ^*##*^*p* < 0.01 vs the control group; *p < 0.05, **p < 0.01 vs the AA group. Low dose: 1 mg/kg/day, high dose: 2 mg/kg/day. Lateral toe volume was measured every 3 days.

After establishing the AA rat model, the rats experienced a CFA-induced secondary inflammatory response of paw swelling, nodules, and redness on the 14th day (Fig. 5A). Compared with that in the AA group, local swelling of AA joints was significantly inhibited in the treatment group on the 32nd day (Fig. 5B). Furthermore, compared with the AA group, the shikonin group (1 and 2 mg/kg) and MTX group (0.5 mg/kg) significantly reduced the degree of foot paw swelling, and the anti-inflammatory effect was more significant in the high-dose group, especially from 20 to 32 days (Fig. 5C). Additionally, shikonin reduced the lactate level in the peripheral blood of AA rats (Fig. 5D). Compared with the control group, there was no significant difference in the degree of damage to the liver and kidney caused by shikonin (Table 1).

**Table 1:** The effect on liver and kidney toxicity in rats.

Group	n	ALT (%)	AST (%)	Cr (%)
Negative group	6	31.7 ± 3.7	135.1 ± 2.8	51.3 ± 4.4
1 mg/kg shikonin	6	31.6 ± 5.4	135.4 ± 4.2	51.6 ± 3.6
2 mg/kg shikonin	6	31.4 ± 4.3	135.4 ± 2.6	51.5 ± 4.5

### Effects of shikonin on the synovial histopathology of AA rats and the expression of Bax, Bcl-2 and cleaved caspase 3 in rat synovial tissue

**Figure 6:**
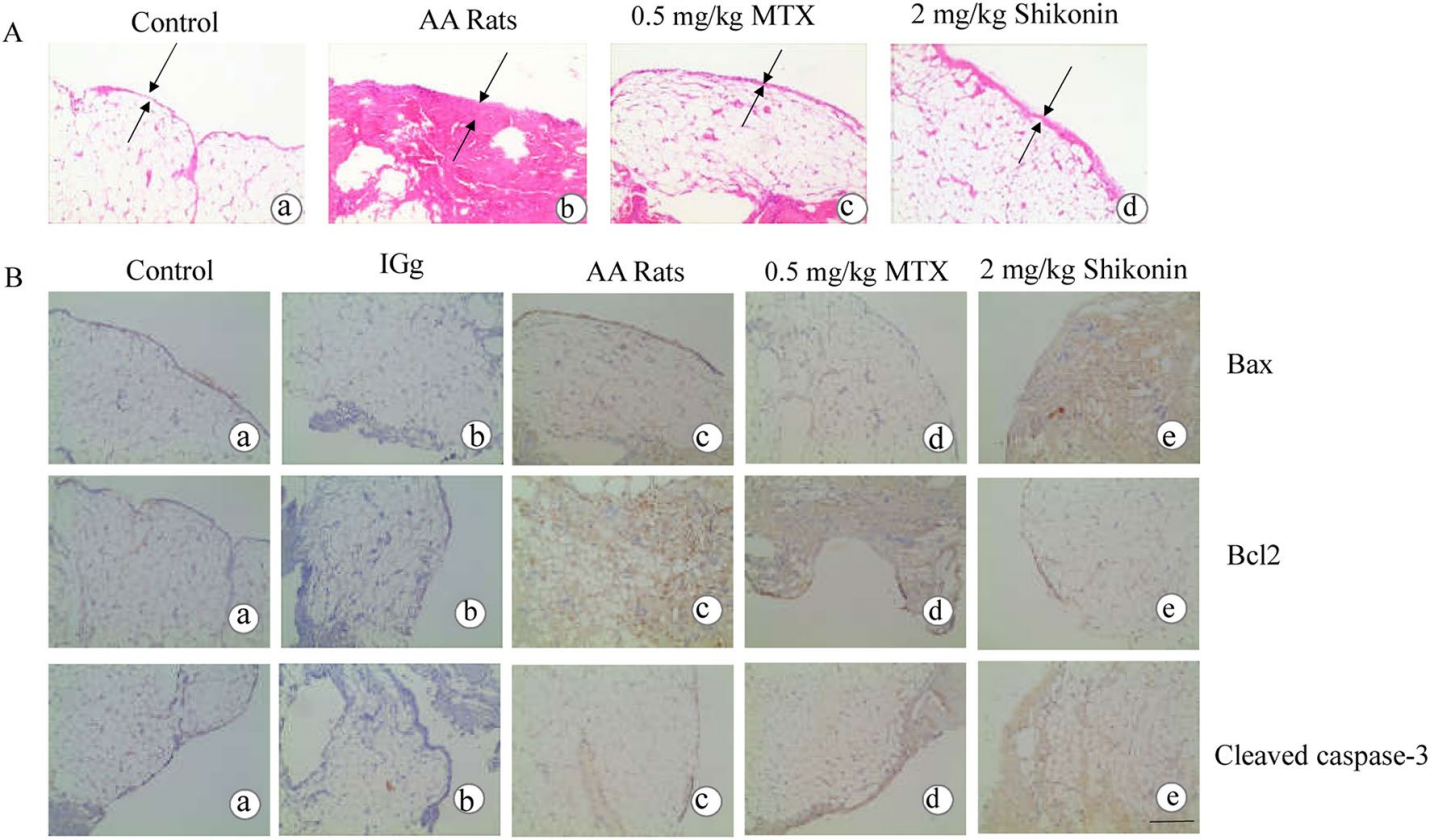
Effects of shikonin on the synovial histopathology of AA rats and the expression of Bax, Bcl-2, and cleaved caspase 3 in rat synovial tissue. (**A**) H&E-stained images of AA rats synovial tissue from the control group, AA group, and shikonin group at 32 days, where black arrows mark synovial hyperplasia. (**B**) Immunohistochemical staining of Bax, Bcl-2, and cleaved caspase 3 in the synovial tissue of AA rats from the control group, IGg group, AA group, and shikonin group. Scale bar represents 200 μm. Data are representative of at least three independent experiments.

To validate the proapoptotic effects of shikonin on RA-FLSs, we evaluated the synovial histopathology of AA rats. Under light microscopy, the cells lining the synovium of the control group were arranged regularly, consisting of 1 to 2 synovial cells stained lightly (Fig. 6A-a). Compared to the control group, the AA group's synovial tissue showed significant proliferation and displayed a disordered cell arrangement and darker staining (Fig. 6A-b). The MTX group and shikonin group showed a lower degree of the abovementioned pathological symptoms (Fig. 6A-c,A-d). To determine whether shikonin can induce synovial tissue damage in AA rats, we evaluated the expression of the apoptosis-related proteins Bax, Bcl-2 and cleaved caspase 3 in the secondary synovial membrane by immunohistochemical staining. The results indicated that the expression levels of Bax and cleaved caspase 3 in the MTX group was higher than those in the control group, and the expression of Bcl-2 was lower in the synovial tissue of AA rats (Fig. 6B).

### Shikonin reduces the levels of TNF-α, IL-6, IL-1β, IL-8, IL-17A, and IL-10 in the serum of AA rats

**Figure 7:**
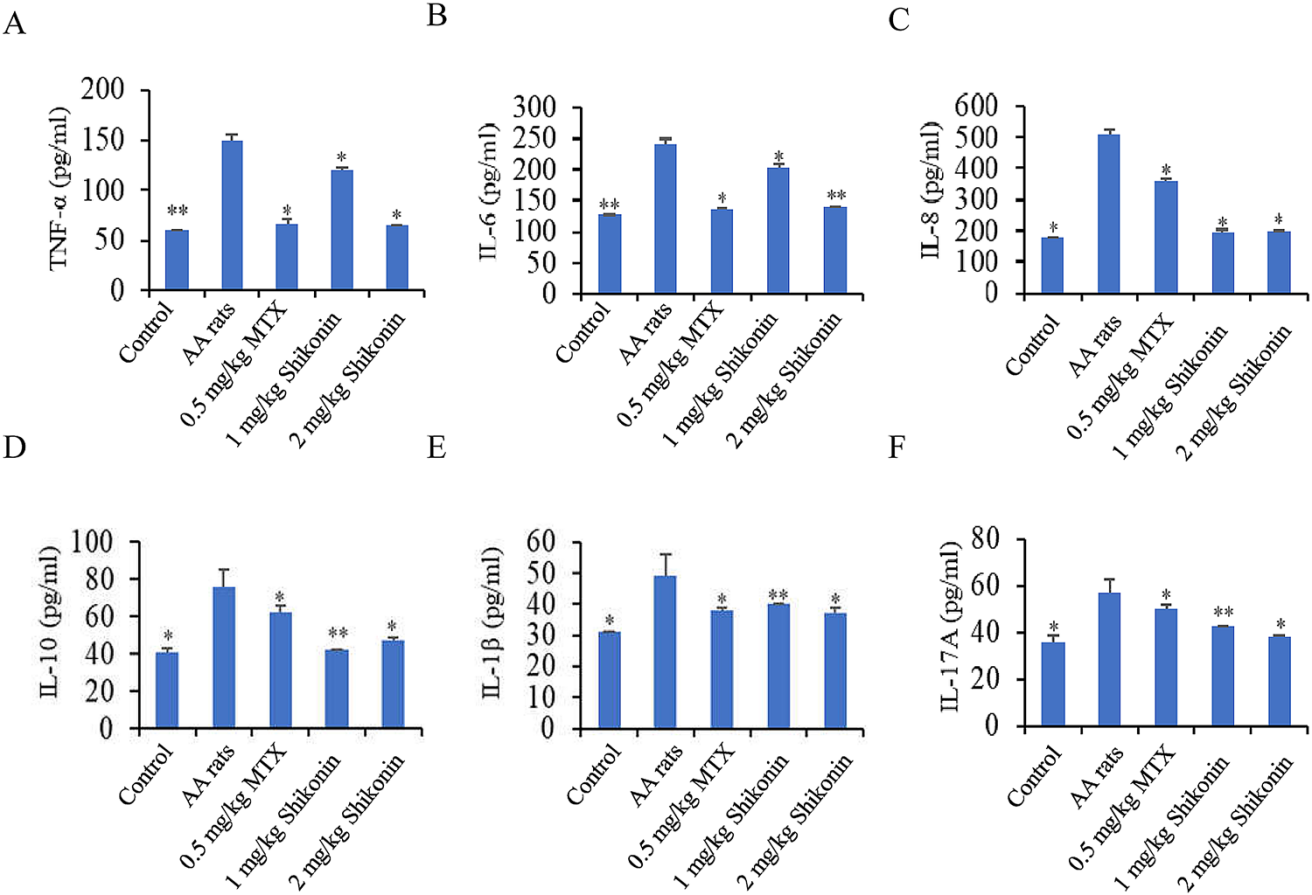
Effects of shikonin on TNF-α, IL-6, IL-8, IL-10, IL-1β, IL-17A levels and release in AA rat serum. (**A**) Serum TNF-α was detected by ELISA. (**B**) Serum IL-6 was detected by ELISA. (**C**) Serum IL-8 was detected by ELISA. (**D**) Serum IL-10 was detected by ELISA. (**E**) Serum IL-1β was detected by ELISA. (**F**) Serum IL-17A was detected by ELISA. *p < 0.05, **p < 0.01, compared to the AA Rats group. Data are representative of at least three independent experiments.

Compared with normal rats, the levels of TNF-α, IL-6, IL-1β, IL-8, IL-17A, and IL-10 in AA rats were significantly increased. Compared with the AA groups, shikonin significantly reduced TNF-α (Fig. 7A), IL-6 (Fig. 7B), IL-8 (Fig. 7C), IL-10 (Fig. 7D), IL-1β (Fig. 7E), and IL-17A (Fig. 7F) levels in serum.

## Discussion

Previous studies have found that RA patients' synovial tissue was hypoxic and accompanied by an increase in glycolytic enzyme gene expression and glycolytic activity^25^. The key enzymes of glycolysis, such as glucose phosphate isomerase (GPI)^26^, aldolase (ALD)^27^, and phosphorylated isomerase (TPI)^28^, can be used as antigens to participate in RA autoimmunity reactions. Additionally, Henderson^29^ used a low-light test and found that the activity levels of glyceraldehyde 3-phosphate dehydrogenase and lactate dehydrogenase, the main pathways, were increased during glycolaldehyde decomposition in RA synovial cells. Curtin^30^ studied the composition of synovial fluid, detected a significant increase in lactic acid content, and decreased glucose concentration in synovial tissue by proton magnetic resonance spectroscopy (MRS), which further confirmed the increase in glycolytic activity. The effect of glucose metabolism on rheumatoid arthritis is mainly reflected in the enhanced cell migration and invasion ability caused by high HK2 expression and the increased extracellular lactic acid level. Regular intraarticular injection of HK2 in the knee could significantly increase the synovial membrane intimal thickness and promote the activation and proliferation of the synovial membrane. HK2 is expressed explicitly in the synovial lining of RA and has a regulatory effect on the severity of arthritis, bone and cartilage damage in mice, and the invasion function of FLS^31^. The microenvironment of the inflammatory joints also appears hypoxic and nutrient deficient^32^, which stimulates a significant increase in glycolysis^33^, mainly by inducing the expression of HIF-1α and GLUT1^34^. Increased expression of GLUT1 leads to an increase in glucose uptake, activation of cell proliferation, and aggravation of the disease course. The penultimate step of glycolysis is catalyzed by pyruvate kinase. PKM2 catalyzes pyruvate production from phosphoenolpyruvate, which is a critical step in ATP production and is essential to energy homeostasis^35^.

Earlier studies have shown upregulation of PKM2 in RA-FLSs, suggesting an essential role of PKM2 in the growth of RA-FLSs and making PKM2 a promising diagnostic indicator^11^. In our study, we found that shikonin can inhibit the expression of intracellular lactate and glycolysis-related enzymes, such as PKM2, GLUT1, and HK2, in RA-FLSs. Shikonin could also inhibit the glycolysis-related PI3K-AKT-mTOR pathway. The pathway regulates many biological activities of cells and participates in cell proliferation, growth, apoptosis, transcription, translation, cytoskeletal rearrangement, and the cell cycle. In addition, PI3K-Akt-mTOR also plays a crucial role in tumor growth, just as it is a major signaling pathway involved in tumorigenesis and development^36,37^. Oncostatin M (OSM) with TNF inhibitors synergistically regulates the cellular bioenergetics and invasive function of synovial cells from patients with rheumatoid arthritis^38^. Hypoxia, glutaminase (GLS)1 and HIF-1α knockdown alters cellular bioenergetics by inducing mitochondrial dysfunction and promoting a switch to glycolysis in human synovial fibroblasts^39–41^.

This study found that while shikonin administration to RA-FLSs led to a sharp decline in intracellular ATP levels while ROS levels increased. The mitochondrial membrane potential of RA-FLSs decreased after 24 h, which confirmed that shikonin could damage mitochondria. The early expression of apoptosis is a decrease in mitochondrial membrane potential. These changes are followed by mitochondrial structural damage, including the release of cytochrome c, apoptosis-inducing factors, and caspase-9 and caspase-3 enzyme-linked reactions^42^. Mitochondria are the primary source of cellular ROS^43^, and abnormal accumulation of ROS may cause oxidative stress and lead to DNA modification and damage^44^. The above results demonstrate that shikonin can inhibit the cellular integrity and functions of RA-FLSs by targeting the mitochondrial ROS pathway.

The transmission electron microscopy results showed that shikonin could induce apoptosis and autophagy in RA-FLSs, as validated by Annexin V-FITC/PI double staining. Compared to the control, the apoptosis level in the shikonin group was significantly higher in RA-FLSs, with minimal apoptosis of FLSs. The mechanism of apoptosis seems to be mediated by Bcl-2, caspase 3 cleavage, and Bax. The ratio of the autophagy marker LC3-II/LC3-I was higher in the shikonin group than in the control group, suggesting that shikonin can promote autophagy in RA-FLSs. Compared with the control group, the mitochondrial swelling, shortening, and vacuolization of RA-FLSs were more obvious in the shikonin group. These data suggest that shikonin can cause significant damage to the mitochondria of RA-FLSs.

In animal experiments, shikonin significantly reduced the degree of swelling of the inflammatory joints in rats. Immunohistochemical analyses indicated that shikonin could inhibit Bcl-2 in rat synovial tissue and promote the expression of Bax. Moreover, we found that shikonin has an excellent inhibitory effect on inflammatory mediators such as TNF-α, IL-6, IL-8, IL-10, IL-17A, and IL-1β and has minimal toxicity to the liver and kidney. In summary, shikonin inhibits the energy metabolism of RA-FLSs by inducing ROS, PI3K-AKT-mTOR, and glycolysis-related proteins to induce apoptosis and autophagy. These data demonstrate that through the mechanisms mentioned above, shikonin can significantly alleviate the degree of swelling and inflammation in the joints and paws in an animal model of RA, thus potentially being a new treatment strategy for rheumatoid arthritis.

## Supplementary Information

Supplementary Information 1.
