# Supplementary material for: Shikonin induces programmed death of fibroblast synovial cells in rheumatoid arthritis by inhibiting energy pathways
Source: Sci Rep. 2021 Sep 14;11:18263. doi: 10.1038/s41598-021-97713-6 (PMC8440543; doi:10.1038/s41598-021-97713-6)
Supplement: Supplementary file 1 — Supplementary Information 1. [file 41598_2021_97713_MOESM1_ESM.pdf]

Shikonin induces programmed death of fibroblast synovial cells in rheumatoid arthritis by inhibiting energy pathways

Jiahui Li[1], Jinglong Pang[1], Zhe Liu[2], XianMing Ge[1], Yanan Zhen[1], Chen Chen Jiang[3,4], Yaming Liu[1], Qiang Huo[1]*, Yiming Sun[2] *, Hao Liu[1]**

[1] School of Pharmacy, Bengbu Medical College, Donghai Road, Bengbu, 233030, Anhui, China.

[2] The First Affiliated Hospital of Bengbu Medical College, Zhihuai Road, Bengbu, 233000, Anhui, China.

[3] Cancer Neurobiology Group, School of Biomedical Sciences & Pharmacy, The University of Newcastle, Callaghan NSW 2308, Australia

[4] Hunter Medical Research Institute, New Lambton NSW 2305, Australia

*Corresponding author: Qiang Huo, School of Pharmacy, Bengbu Medical College, Anhui, China, 620793985@qq.com

*Corresponding author: Yiming Sun, The First Affiliated Hospital of Bengbu Medical College, Anhui, China, 15951977608@163.com

**Corresponding author: School of Pharmacy, Bengbu Medical College, Anhui, China, liuhao6886@foxmail.com

Jinglong Pang: 834752819@qq.com

XianMing Ge: 1554261819@qq.com

Yanan Zhen: 984863513@qq.com

Yaming Liu: 651667143@qq.com

Zhe Liu: 117915493@qq.com

Supplementary 1

Table 1: All the antibody for WB

Antibody name	Molecular Weight (KDa)	Species	Reagent Company
PKM2	60	Rabbit	Cell signaling technology (Shanghai)
GLUT1	54	Rabbit	Abcam (Shanghai) Trading Co., Ltd.
HK2	102	Rabbit	Abcam (Shanghai) Trading Co., Ltd.
AKT	56	Rabbit	Abcam (Shanghai) Trading Co., Ltd.
p-AKT	60	Rabbit	Abcam (Shanghai) Trading Co., Ltd.
PI3K	111	Rabbit	Abcam (Shanghai) Trading Co., Ltd.
p-PI3K	85	Rabbit	Abcam (Shanghai) Trading Co., Ltd.
mTOR	289	Rabbit	Abcam (Shanghai) Trading Co., Ltd.
Bax	21	Rabbit	Abcam (Shanghai) Trading Co., Ltd.
Bcl-2	26	Rabbit	Abcam (Shanghai) Trading Co., Ltd.
caspase-3	35, 17	Rabbit	Abcam (Shanghai) Trading Co., Ltd.
LC3	16, 14	Rabbit	Abcam (Shanghai) Trading Co., Ltd.
β-actin	43	Rabbit	Proteintech (Wuhan)

Supplementary 2

Table 2 : Primers used for real-time PCR

Genes	Forward (5′–3′)	Reverse (5′–3′)
GLUT1	CCCAGCAGCAAGAAGCTGAC	GGGGCATTGATGACTCCAGTG
PKM2	TTCCAGATCAGGCGGCTCTCC	GGCTGCTGAGGTCCTTTGGTTC
PKLr	GCTTTGCAGGTTCCCCACTCAG	TCACCAGCCCACTTCCGACTC
HK2	CGCTGGCAACATCGTGCACC	CTCGGGCAGTGGGACCTCTC
Pgam2	GAGTCCTCTGTGGTCCCTGCTG	GTTCTCCTGGTTCCATGTGCTCT
Pfkm	GTCAGGGCTGTGGTTCGAGTTG	GGTGGCTTCCTTGATGTGATCTCC
Acss2	GAACTTGACGTGATGGGGCTTCC	CCTCCGGCTCCAGCTTCCTC
GAPDH	CAGGAGGCATTGCTGATGAT	GAAGGCTGGGGCTCATTT

Supplementary 3

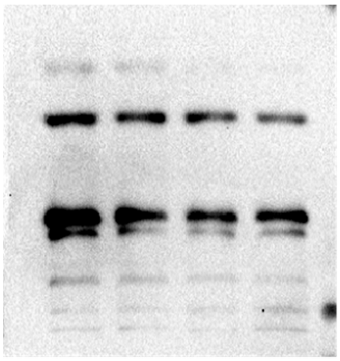

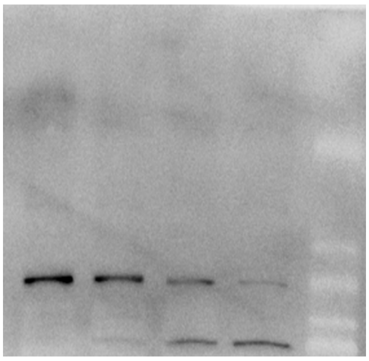

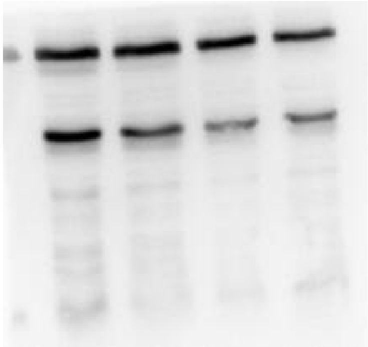

Fig-1-F-PKM2

Fig-1-F-GLUT1

Fig-1-F-HK2

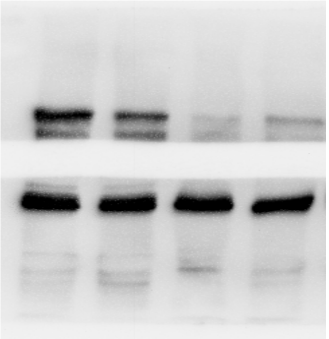

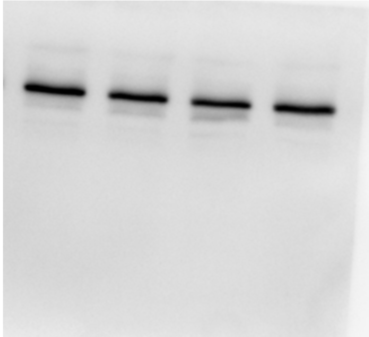

Fig-1-F-PI3K-and-p-PI3k

Fig-1-F-AKT

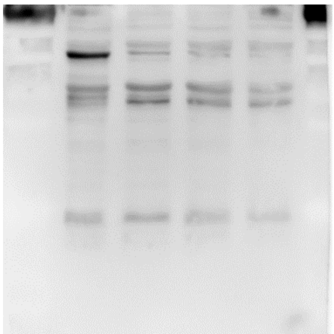

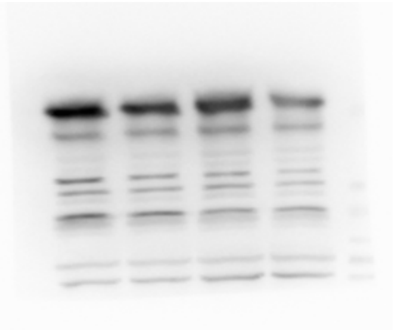

Fig-1-F-p-AKT

Fig-1-F-mTOR

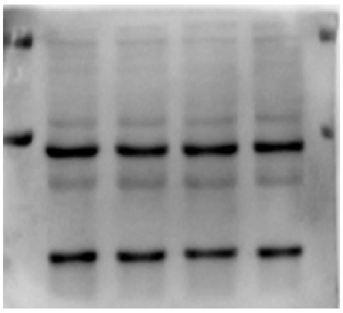

Fig-1-F-β-action

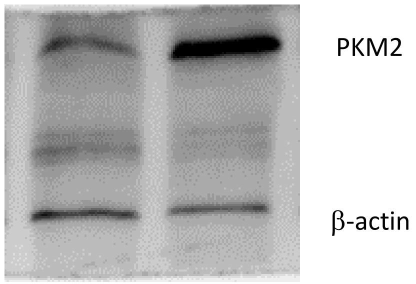

Fig-2-A

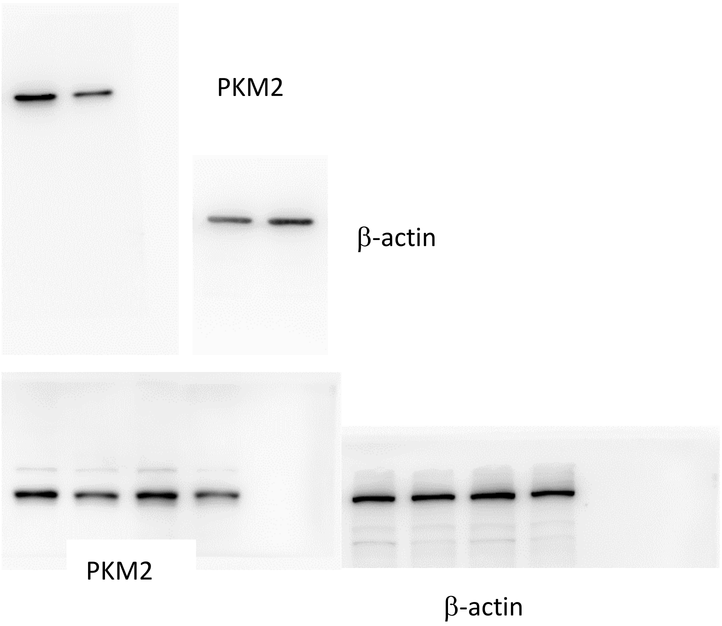

Fig-2-B

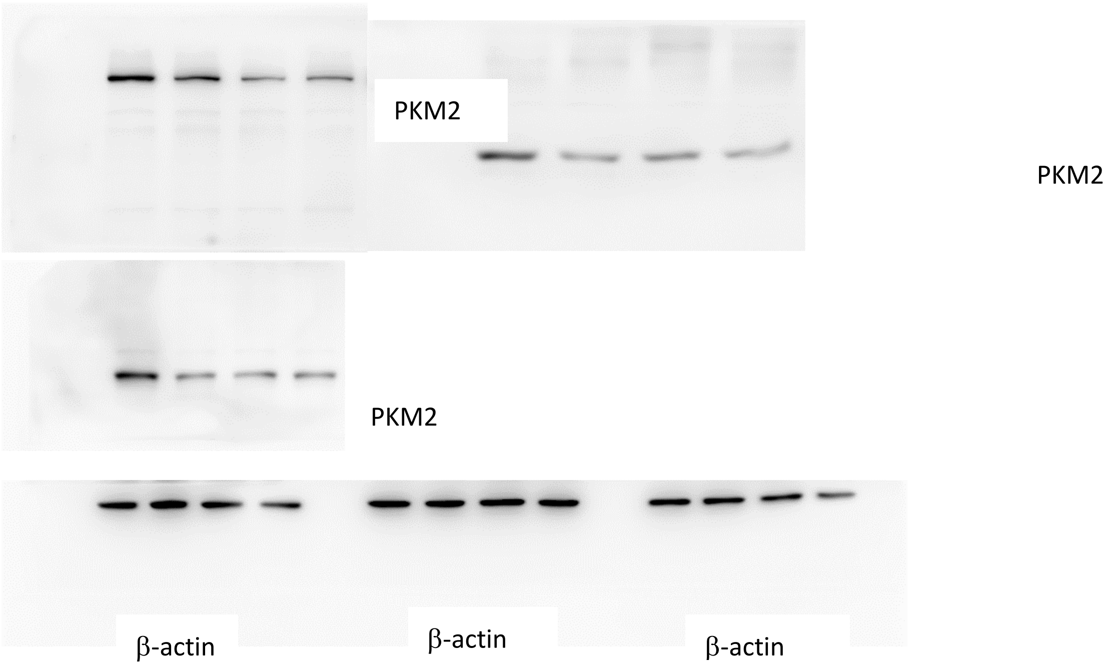

Fig-2-C

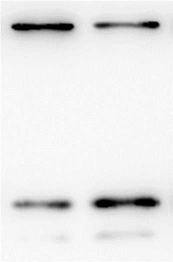

Tetramer

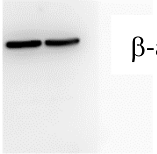

β-actin

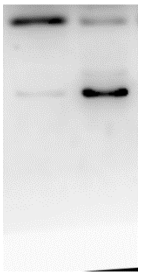

Tetramer

β-actin

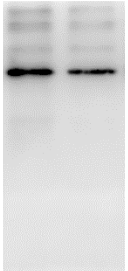

PKM2

Fig-2-E

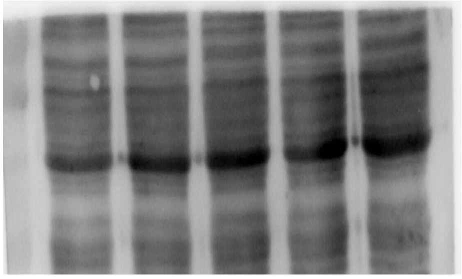

Fig-3-E-Bax

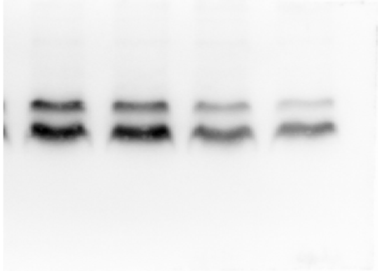

Fig-3-E-Bcl2

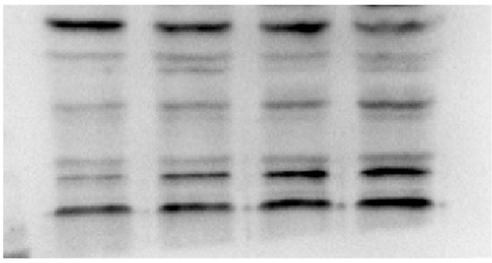

Fig-3-E-Cleaved-casepase-3

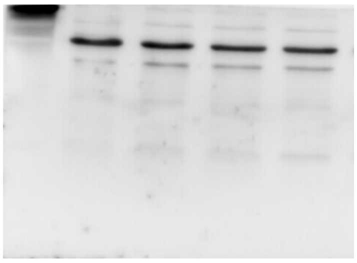

Fig-3-E-β-action

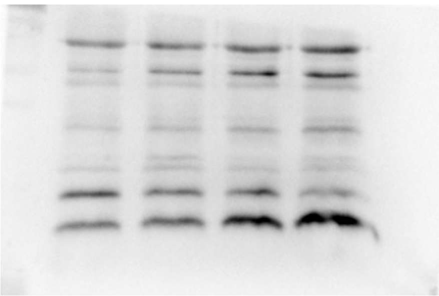

Fig-4-C-LC3II

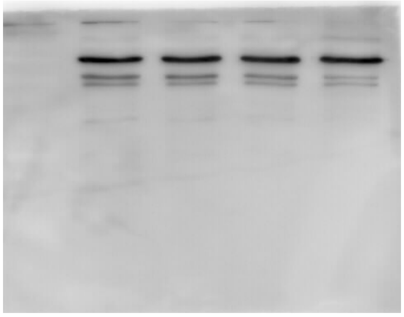

Fig-4-C-β-action
